# To the Editors of the Medical and Physical Journal

**Published:** 1810-08

**Authors:** John Taunton

**Affiliations:** Greville Street, Hatton Garden


### To the Editors of the Medical and Physical Journal.

GENTLEMEN,

IN the last Number of your Journal, I see that you have inserted a Report from the National Vaccine Establishment, which differs so widely from my observations on that subject, that I have been induced to hand you the following statement, of which you are at liberty to make what use you please.

In the Finsbury Dispensary, St. John's Street, Clerkenwell, it has been my practice for the last four years, to vaccinate the patients, or to inoculate them for the small-pox, agreeably to their own choice; nor have any means been taken to bias their judgment; within the last twelve months, only four patients have been vaccinated, and upwards of one thousand have been inoculated for the small-pox.

I am, &c,

JOHN TAUNTON.

*Greville Street, Hatton Garden,*
*July* 4, 1810.

